# Female sexual dysfunction in multiple system atrophy: a prospective cohort study

**DOI:** 10.1007/s10286-021-00825-2

**Published:** 2021-09-07

**Authors:** Cecilia Raccagni, Elisabetta Indelicato, Victoria Sidoroff, Martin Daniaux, Angelika Bader, Bettina Toth, Lukas A. Jelisejevas, Margarethe Hochleitner, Alessandra Fanciulli, Fabian Leys, Sabine Eschlboeck, Christine Kaindlstorfer, Sylvia Boesch, Gregor K. Wenning

**Affiliations:** 1 Department of Neurology, Medical University of Innsbruck, Anichstrasse 35, 6020 Innsbruck, Austria; 2 Department of Neurology, Regional General Hospital, Lorenz Boehler Strasse 5, 39100 Bolzano, Italy; 3 Center for Rare Movement Disorders Innsbruck, Medical University of Innsbruck, Anichstrasse 35, 6020 Innsbruck, Austria; 4 Department of Radiology, Medical University of Innsbruck, Anichstrasse 35, 6020 Innsbruck, Austria; 5 Gender Medicine and Diversity Unit, Medical University of Innsbruck, Innrain 66, 6020 Innsbruck, Austria; 6 Department of Gynecological Endocrinology and Reproductive Medicine, Medical University of Innsbruck, Anichstrasse 35, 6020 Innsbruck, Austria; 7 Department of Urology, Medical University of Innsbruck, Anichstrasse 35, 6020 Innsbruck, Austria

**Keywords:** Multiple system atrophy, Non-motor symptoms, Autonomic failure, Sexual dysfunction, Female, Arousal, Lubrication

## Abstract

**Purpose:**

The diagnosis of probable multiple system atrophy relies on the presence of severe cardiovascular or urogenital autonomic failure. Erectile dysfunction is required to fulfil the latter criterion in men, whereas no corresponding item is established for women. In this study, we aimed to investigate sexual dysfunction in women with multiple system atrophy.

**Methods:**

We administered the Female Sexual Function Index questionnaire and interviewed women with multiple system atrophy and age-matched controls regarding the presence of “genital hyposensitivity.”

**Results:**

We recruited 25 women with multiple system atrophy and 42 controls. Female Sexual Function Index scores in sexually active women with multiple system atrophy were significantly lower (multiple system atrophy = 10; 15.4, 95% CI [10.1, 22.1], controls = 37; 26.1 [24.1, 28.1], *p* = 0.0004). The lowest scores concerned the domains of desire, arousal and lubrication. Genital hyposensitivity was reported by 56% of the patients with multiple system atrophy and 9% controls (*p* < 0.0001).

**Conclusions:**

Sexual dysfunction is highly prevalent in women with multiple system atrophy. Screening for disturbances in specific sexual domains should be implemented in the clinical evaluation of women with suggestive motor symptoms.

## Introduction

Autonomic dysfunction is a cardinal feature of synucleinopathies, and it is most severe in multiple system atrophy (MSA), a progressive, fatal disorder, which features a combination of parkinsonian and/or cerebellar motor signs along with autonomic failure [1]. The clinical diagnosis of MSA at a probable level of certainty requires the presence of severe cardiovascular or urogenital autonomic dysfunction [1]. According to consensus criteria, erectile dysfunction in addition to urinary incontinence is required to fulfil the urogenital dysfunction criterion in male patients [1]. Currently, there is no equivalent item for female patients.

While genital disorders are well described for male patients with MSA [2–5], there is a lack of information about sexual symptoms in women with MSA. Only one study has evaluated sexual function in women with the parkinsonian variant of MSA (MSA-P), investigating the presence of “genital hyposensitivity” [6]. Instruments which explore sexual function in female patients with MSA are virtually nonexistent, being limited to one dedicated question on the MSA-specific Unified Multiple System Atrophy Rating Scale (UMSARS) questionnaire [7]. Moreover, information about sexual dysfunction in female patients with MSA of the cerebellar type (MSA-C) is lacking.

In the present study, we investigated sexual function by means of a standardized questionnaire, the Female Sexual Function Index (FSFI), in female patients with MSA and in age-matched controls, as well as the effect of mood and concomitant gynecological comorbidities. We aimed to characterize sexual dysfunction in women with MSA and to identify aspects of sexual dysfunction which may hint at a diagnosis of MSA in female patients.

## Methods

This study was approved by the local ethics committee (study number: 1232/2017). Written informed consent was given by the patients and controls prior to inclusion. Female patients with a diagnosis of probable or possible MSA-P or MSA-C according to the current diagnostic criteria [1] were recruited at the Neurology Department of the Medical University of Innsbruck. Age-matched female controls were recruited from (1) women referred for screening breast sonography/mammography and (2) women referred for a screening health examination at our university hospital. Exclusion criteria consisted of dementia or clinically evident major cognitive impairment, major depressive or psychotic disorder, presence of other organic causes of dysautonomia (included but not limited to diabetes, toxic or metabolic peripheral neuropathy, presence of other genital confounders as per investigator judgement), and current estrogen replacement or antiestrogenic therapy.

The severity of motor and non-motor symptoms in patients with MSA was assessed using the UMSARS [7]. One item of the UMSARS asks patients to rate their sexual function “compared to healthy days,” with possible scores ranging from 0 (= no impairment) to 4 (= no sexual activity possible).

Patients and controls were specifically interviewed about the presence of genital hyposensitivity (key question: “do you experience a loss of genital feeling during sexual intercourse?”) and, if applicable, about its onset and duration. To further investigate sexual dysfunction, we administered the German version of the FSFI [8], a self-report questionnaire that was developed to assess different domains of sexual function in women (desire, arousal, lubrication, pain, orgasm, satisfaction). Each domain is covered by 2–4 questions for a total of 19 items over the 4 weeks preceding the examination. The score ranges from 2 (no sexual dysfunction) to 36 (maximal level of sexual dysfunction). Detailed explanations were given by the recruiting physician before compilation. In order to guarantee confidentiality, a second study team member collected the patients’ questionnaires and extracted data.

The Beck Depression Inventory-II (BDI-II) was used to investigate the participants’ mood.

We performed statistical analysis using SPSS version 25 software. For the comparative analysis of FSFI scores, we applied analysis of covariance (ANCOVA) to be able to consider covariates. Since the FSFI scores remained non-normally distributed after logarithmic and Box-Cox automated data transformation, bias-corrected and accelerated bootstrapping with 1000 iterations was applied to account for non-normality. Statistical significance was set at *p* < 0.05.

## Results

We recruited 25 women with MSA and 42 age-matched female controls (see Table 1). All study participants were living at home. All study participants but one control (bisexual) reported to be heterosexual.

**Table 1:** Clinical data for female patients with MSA

	MSA (*n* = 25)
Subtype	13 MSA-P, 12 MSA-C
Level of diagnostic certainty	21 probable, 4 possible
Disease duration (years)	5 [3, 6]
UMSARS sum score	42 [33, 50]
Orthostatic hypotension (*n*,%)	18 (72%)
Urinary disturbances (*n*, %)	20 (80%)
UMSARS sexual item score	2.5 [1.7, 3.2]
0 = No problems	6 (24%)
1 = Minor impairment compared to healthy days	0
2 = Moderate impairment compared to healthy days	2 (8%)
3 = Severe impairment compared to healthy days	6 (24%)
4 = No sexual activity possible	8 (32%)
No answer	3 (12%)

Data are reported as percentage or mean and 95% [confidence interval]

Women with MSA were more frequently sexually non-active (*n* = 15, 60%, vs. *n* = 5, 12% in controls, *p* < 0.0001). Disease duration did not differ between sexually active and non-active patients, but the latter were more severely affected, as reflected by the UMSARS sum scores (50, 95% CI [37.1, 63.6] in sexually non-active vs. 29.3 [24.3, 34.4] in sexually active patients, *p* = 0.03).

The FSFI questionnaire was collected in sexually active participants (MSA = 10, controls = 37). FSFI sum scores in women with MSA were significantly lower (15.9, 95% CI [10.1, 22.1] vs. 26.2 [24.1, 28.1] in controls, *p* = 0.0004 after adjusting for age and BDI-II scores). Across the FSFI sub-items, the domains “desire,” “lubrication” and “arousal” displayed the lowest scores (see Table 2). FSFI scores and scores at the UMSARS sexual item showed no correlation [*r*(8) = 0.1, *p* = 0.74]. There were also no significant correlations between the FSFI sum scores/subscores and total UMSARS scores, UMSARS I and II subscores or with disease duration. Considering the entire study collective, FSFI sum scores showed a moderate correlation with BDI-II scores [*r*(42) = −0.51, *p* = 0.001 two-tailed]. Postmenopausal women displayed a tendency towards lower FSFI sum scores (23.4, 95% CI [20.7, 26.2] vs. 26.5, [23.1, 29.9] in premenopausal women, *p* = 0.6) (Table 3).

**Table 2:** Comparison between women with MSA and controls

	MSA (*n* = 25)	Controls (*n* = 42)	*p* value
Age at examination (years)	60 [57, 62]	58 [55, 60]	0.2
Menopausal status (*n*, %)	21 (82)	34 (81)	0.5
Gynecological disorders (*n*, %)	6 (24)	14 (33)	0.3
Sexual inactivity (*n*, %)	15 (60)	5 (12)	< *0.0001*
BDI-II score	15 [12, 18]	11 [7, 15]	*0.01*

FSFI	MSA (*n* = 10)	Controls (*n* = 37)	*p* value
Sum score	15.4 [10.1, 22.1]	26.1 [24.1, 28.1]	*0.0004*
Desire	2.2 [1.5, 3.1]	3.5 [3.1, 3.8]	*0.01*
Arousal	3 [1.8, 4.2]	4.5 [4.1, 4.9]	*0.02*
Lubrication	3.2 [2.2, 4.1]	4.5 [4, 4.9]	*0.03*
Orgasm	3 [1.6, 4.4]	4.1 [3.6, 4.6]	0.2
Satisfaction	4.5 [3.5, 5.4]	4.9 [4.6, 5.2]	0.6
Pain	4.9 [4, 5.7]	4.7 [4.1, 5.2]	0.4

Data are reported as percentage or mean and 95% [confidence interval]. Statistically significant results are marked in bold

**Table 3:** Gynecological disorders in female patients with MSA and controls

Gynecological disorders	
Controls	MSA
9 Hysterectomy	2 Hysterectomy
2 Breast cancer	2 Vaginal banding/colporrhaphy
1 Ovariectomy	1 Breast cancer
1 Endometriosis	1 Status post implantation of a suprapubic catheter
1 Uterus myomatosus	

Women with MSA had a higher prevalence of genital hyposensitivity (*n* = 14, 56% vs. *n* = 4, 9% in controls, *p* < 0.0001). Across the MSA group, 13 out of 19 patients with probable MSA and one out of four with possible MSA described genital hyposensitivity. This disturbance was equally distributed between MSA-P and MSA-C. No relationship was found between hyposensitivity and presence of urinary disturbances (*p* = 0.8). Genital hyposensitivity was not associated with presence/absence of sexual activity (*p* = 1). Four women with MSA could describe when they first noticed genital hyposensitivity. Anorgasmia and genital hyposensitivity were present at the onset of the disease in two of them, appeared within 2 years from motor onset in a further woman, and in a fourth developed after 9 years. Considering the entire study collectively, genital hyposensitivity was associated with lower FSFI scores (14.5, 95% CI [9.2, 19.5] vs. 23.8 [20.8, 26.7] in subjects without hyposensitivity, *p* = 0.02 after adjusting for age and BDI-II scores). In women with MSA, genital hyposensitivity was strongly associated with a lower rating on the UMSARS sexual item (*p* = 0.00002).

## Conclusion

The present study addressed the characterization of sexual function in women with MSA, an issue which has been neglected by the clinical research in the field [1].

Our findings show that sexual dysfunction is highly prevalent in female patients with MSA, regardless of the motor subtype. The application of a standardized, female-specific scale quantified the sexual dysfunction in women with MSA as significantly more severe than that in age-matched controls. The most commonly affected sexual domains in MSA as compared to controls were desire, arousal and lubrication.

Over the past decade, cumulative evidence has highlighted autonomic failure as a relevant diagnostic and prognostic marker in synucleinopathies. In particular, erectile dysfunction may be an early sign, affecting nearly all men with MSA [2–4, 9]. The presence of isolated erectile dysfunction enables a diagnosis of possible MSA and is even required, in addition to urinary incontinence, to diagnose probable MSA in male patients. In contrast, there is no such item defined for female patients, implying a poorer diagnostic yield of current criteria in women. The only study addressing this issue dates back to 2003. Oertel and colleagues investigated “genital sensitivity” in 19 women with MSA-P and showed that 47% had reduced sensitivity of the clitoris which appeared in close temporal correlation with disease onset [6].

Sexual dysfunction is a highly prevalent symptom in chronic illnesses [10]. Nonetheless, during clinical encounters, inquiry about sexual complaints is often omitted [10], and women in particular are not likely to bring up the problem if they are not asked directly [11]. Beyond this issue, the lack of clearly defined endpoints and outcomes has caused research on female sexual dysfunction to lag behind that of males [8]. Furthermore, the mean age of onset in MSA overlaps with menopause, an additional factor which hampers the definition of a clear-cut marker of sexual autonomic dysfunction, due to the high prevalence of gynecological confounders.

The FSFI allowed us to investigate the distribution of disease burden among the different domains of sexuality, revealing that “desire,” “lubrication” and “arousal,” in descending order, were the areas most highly affected in women with MSA as compared to controls. While central components of sexuality, such as “desire” or “satisfaction,” are strongly influenced by mood, other domains are more prone to reflect organic disturbances. Arousal involves the activation of central and peripheral mechanisms that coordinate the sexual act [12]. Genital arousal results in penile erection in males and in vaginal lubrication and congestion of the clitoris and vulva in females [12]. Apart from the anatomical differences, the same neurovascular and biochemical mechanisms underlie these processes in women and men [12]. Notable, a neuropathological hallmark of MSA is the degeneration of Onuf's nucleus, which innervates the key pelvic muscles involved in genital arousal in both sexes [13]. Thus, our findings reveal a pattern of impairment of female sexuality—arousal and lubrication—which corresponds to that of men with MSA. Despite the greater influence of psychological factors in female sexuality, this parallel underpins a relevant dysautonomic aspect of sexual dysfunction in women with MSA. In turn, these extensive changes in genital innervation culminate in the loss of reactivity to stimuli, which is described as “reduced genital sensitivity.” Intriguingly, patients and controls displayed similar scores concerning the domain “pain.” Since reduced lubrication and arousal may lead to dyspareunia [14], it could be expected that patients would experience pain during sexual intercourse more frequently than controls. In contrast to this assumption, our results may rather reflect the reduced genital sensitivity which affects women with MSA.

Sexual inactivity was highly prevalent among women with MSA in our study. We did not interview patients about the underlying causes, which are most likely multifactorial, with neurological disability, sexual dysfunction and psychological factors all contributing. Dissecting the contribution of these components is arduous. This issue represents in our opinion a major drawback of the current gender-nonspecific evaluation based on the single question on the UMSARS. For instance, the answer “no sexual activity possible” at UMSARS may be due to physical limitations rather than anorgasmia in women.

Our study had a pilot design, and the small patient group reflects the rarity of the disease and the recruitment of one gender, but also the strict inclusion criteria applied in the study. In this preliminary study, we did not aim to evaluate sexual dysfunction in related disorders (Parkinson's disease and sporadic adult-onset ataxia of unknown etiology). We did not investigate the single domain of sexual dysfunction specifically or address its pathophysiological basis. Importantly, we also did not explore the correlation between urological autonomic failure and sexual disorder. Nonetheless, we investigated for the first time the pattern of sexual dysfunction in female patients with MSA by means of a validated, dedicated, female-specific instrument. Based on the present findings, we endorse the application of a semi-structured interview including (1) disturbances of genital arousal and lubrication and (2) time of onset of symptoms, along with (3) a short gynecological history in women with suggestive motor symptoms. The application of these items in natural history studies, as a female counterpart to surveys on erectile dysfunction, may lead to the implementation of clinical diagnostic criteria for MSA in women.

## Declarations

### Data Availability

Raw data are available upon request.
